# Gradient Porous Structured MnO_2_-Nonwoven Composite: A Binder-Free Polymeric Air Filter for Effective Room-Temperature Formaldehyde Removal

**DOI:** 10.3390/polym14122504

**Published:** 2022-06-20

**Authors:** Zijian Dai, Jianyong Yu, Yang Si

**Affiliations:** 1 State Key Laboratory for Modifcation of Chemical Fibers and Polymer Materials, College of Materials Science and Engineering, Donghua University, Shanghai 201620, China; zjdai@dhu.edu.cn; 2 Innovation Center for Textile Science and Technology, Donghua University, Shanghai 200051, China

**Keywords:** MnO_2_, bicomponent polyolefin nonwovens, polymeric filter, binder-free, HCHO removal

## Abstract

Recently, MnO_2_-coated polymeric filters have shown promising performance in room-temperature formaldehyde abatement. However, a commonly known concern of MnO_2_/polymer composites is either MnO_2_ crystal encapsulation or weak adhesion. This work reports a low-cost high-throughput and green strategy to produce binder-free MnO_2_-nonwoven composite air filters. The production approach is energy saving and environmentally friendly, which combines MnO_2_ crystal coating on bicomponent polyolefin spunbond nonwovens and subsequent heat immobilizing of crystals, followed by the removal of weakly bonded MnO_2_. The binder-free MnO_2_-nonwoven composites show firm catalyst-fiber adhesion, a gradient porous structure, and excellent formaldehyde removal capability (94.5% ± 0.4%) at room temperature, and the reaction rate constant is 0.040 min^−1^. In contrast to the MnO_2_-nonwoven composites containing organic binders, the HCHO removal of binder-free filters increased by over 4%. This study proposes an alternative solution in producing catalyst/fabric composite filters for formaldehyde removal.

## 1. Introduction

Formaldehyde (HCHO), one of the major indoor air pollutants, is reported to be continuously emitted from construction and decoration materials [1,2]. In 2004, the International Agency for Research on Cancer (IARC) classified HCHO as carcinogenic to humans (Group 1), as evidence shows that nasopharyngeal cancer and leukemia correlate with exposure to formaldehyde [3,4]. In 2010, the World Health Organization (WHO) set the indoor guideline value for HCHO as 0.1 mg/m^3^, which has been adopted by major countries, such as Australia, China, Germany, and Japan [5]. According to a recent study investigating the residential HCHO concentrations in nine provinces in China, almost half (45.7%) of the residences exceeded the WHO limit [6]. Therefore, developing universal room-temperature HCHO treatment approaches is important and welcomed [7,8,9]. The commonly used catalysts for HCHO abatement include noble metals [10,11], transition metal oxides [12,13,14], modified carbons [15,16], metal–organic frameworks [17,18,19], and so on; however, transition metal oxide is the most attractive heterogeneous catalyst in terms of robust performance and low energy consumption.

Among transition metal oxide catalysts, manganese dioxide (MnO_2_) is widely used in HCHO heterogeneous catalytic removal as it can readily oxidize HCHO and convert it to H_2_O and CO_2_ [20,21,22]. However, MnO_2_ crystals are difficult to handle and utilize, not ideal for room-temperature HCHO abatement in practical applications [23]. In this regard, immobilizing MnO_2_ on a fibrous substrate is of great interest, because the firm adhesion of MnO_2_ on substrates sets a fundamental basis for real applications.

Previous reports on MnO_2_-fiber composite filters studied their performance for HCHO removal at room temperature. For example, Wang et al. [24] developed a one-step hydrothermal approach to deposit MnO_2_ on polyethylene terephthalate (PET) fibers, allowing over 94% removal efficiency of HCHO (0.6 mg cm^−3^) at room temperature under a gas hourly space velocity (GHSV) of 90 L g_cat_^−1^ h^−1^. Hu et al. [25] produced a MnO_2_/polystyrene fibrous structure. Using 5 ppm of HCHO and a GHSV of 60 L g_cat_^−1^ h^−1^, the composites enable up to 88.2% HCHO removal efficiency. Additionally, Qu et al. [26] recently reported that utilizing a friction-heating adhesion approach shortened the production time of MnO_2_@PET composite. A possible concern with these systems is the low throughput in production, which could add cost to the final product. Organic binders are usually used to bind powderlike catalysts with fibrous substrates at mills. Sekine et al. [27] demonstrated continuous HCHO degradation capability using a fiber cloth filter binding with MnO_2_ and activated carbons. However, the catalytic oxidation performance was partially hampered by the organic binders [24]. Besides, binders may also pose a threat to body health in production and utilization [28].

Direct adhesion of MnO_2_ on polymeric fabrics remains a challenge for a scalable process. Therefore, it is of great importance to develop a green method that produces catalyst-supported hybrid filters at lower cost and high throughput. Here, we proposed a robust methodology to produce MnO_2_-coated fibrous filters by attaching MnO_2_ to bicomponent polyolefin (bico-polyolefin) spunbond nonwovens. The bico-polyolefin spunbond nonwovens are made with core component polypropylene (PP) surrounded by sheath component polyethylene (PE). Due to its different melting temperature, a PE sheath will be sufficiently softened and infused to bind MnO_2_ crystals at a lower temperature without destroying PP cores; thus, no additional organic binder is required for the system. The binder-free MnO_2_-nonwoven composite filters have gradient pore structures and excellent catalytic oxidation ability of HCHO at room temperature.

## 2. Materials and Methods

### 2.1. Materials

Potassium permanganate (≥99.5%, KMnO_4_) was supplied by Shanghai Lingfeng Chemical Reagent Co., Ltd., Shanghai, China. n-Butyl alcohol (≥99.5%, BA) was supplied by Shanghai Titan Scientific Co., Ltd., Shanghai, China. Poly(vinyl alcohol) (PVA) 1788 and HCHO solution (≥36%) were purchased from Shanghai Aladdin Reagent Co., Ltd., Shanghai, China. All reagents were of analytical grade and used without further purification. Hydrophilic bicomponent polyolefin (polyethylene/polypropylene, PE/PP) nonwovens with a basis weight of 110 g m^−2^ were provided by Hangzhou Holyway Co., Ltd., Hangzhou, China. Styrene–acrylic emulsion (SAE, 7199A, solid content, 48.2%) was purchased from Guangzhou Suixin Chemical Co., Ltd., Guangzhou, China.

### 2.2. Synthesis of MnO_2_ Catalysts

MnO_2_ crystals were synthesized using reported procedures [29]. Briefly, 37.92 g of KMnO_4_ was fully dissolved in 600 mL of distilled water under vigorous stirring at room temperature. Then 96 mL of BA was added to the above solution and stirred constantly for 12 h to complete the redox reaction. The synthesized crystals were filtered and washed in deionized water twice, finally dried at 90 °C for 12 h before use.

### 2.3. Preparation of MnO_2_-Nonwoven Composites

**Figure 1:**
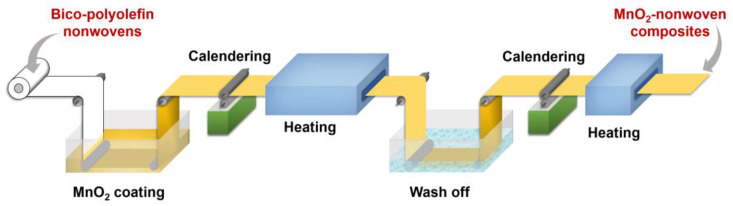
Technological production process of MnO_2_-nonwoven composite filters.

To produce binder-free MnO_2_-nonwoven composite air filters, a laboratory production line was utilized, as depicted in Figure 1. First, a certain amount of MnO_2_ crystals was added to the water tank. After this step, PVA solution was added to the above water tank, which allowed MnO_2_ crystals to suspend in solution. The weight percentage of the PVA agent was controlled at 1–3% compared with MnO_2_. Then the precursor solution was under vigorous stirring for 10 h to create MnO_2_ suspension. After that, one piece of bico-polyolefin nonwoven swatch (11 cm × 12 cm) was soaked into MnO_2_ suspension, and then heated at 135 °C for 3 min after going through the calendering process. When the heating process was completed, the MnO_2_-nonwoven composite was washed in a water bath to remove PVA, unbonded MnO_2_, and was finally dried at 100 °C (Figure S1). Repeating the above soaking, calendering, heating, and washing steps one more time, the binder-free MnO_2_-nonwoven composite was obtained. Using 10%, 15%, and 20% of MnO_2_ concentration in solution, the loading amount of MnO_2_ in composite air filters ranges from 45 to 66 wt% (Table 1), denoted as 10%MnO_2_@Polyolefin, 15%MnO_2_@Polyolefin, and 20%MnO_2_@Polyolefin, respectively. The uniform dark color of the as-prepared MnO_2_-nonwoven composite confirms full coverage of white colored bico-polyolefin spunbond nonwovens (Figure S2).

As control, another type of MnO_2_-nonwoven composite was produced, where MnO_2_ was bonded to PE/PP nonwovens with a SAE binder, denoted as MnO_2_@binder@Polyolefin. To prepare MnO_2_@binder@Polyolefin, 15% of MnO_2_ crystals were first added to the water tank and stirred. Then SAE (SAE/MnO_2_, 0.4:1, *w*/*w*) was added under stirring. After that, another PE/PP swatch (11 cm × 12 cm) was soaked into the above solution, calendered, and heated at 90 °C for 5 min.

### 2.4. Characterization

X-ray diffraction (XRD) pattern was recorded on an XRD tool (D/max-II B, Shimadzu, Kyoto, Japan) to investigate the crystalline structure. Fourier-transform infrared (FTIR) spectroscopy was carried out with a spectrometer (Nicolet iS10, Thermo Fisher, Waltham, MA, USA). The surface morphology of bico-polyolefin spunbond nonwovens and MnO_2_-nonwoven composites was investigated using a scanning electron microscope (SEM, SU5000, Hitachi, Tokyo, Japan). The pore size distributions of PE/PP nonwovens and MnO_2_-nonwoven composites were evaluated by utilizing a capillary flow porometer (CFP-1100AI, Porous Materials, Inc., Ithaca, NY, USA). The pressure drop of the bico-polyolefin nonwovens and MnO_2_-nonwovens composites was measured by employing an automated filter tester (TSI 8130, TSI, Inc., Shoreview, MN, USA).

The formaldehyde (HCHO) removal efficiency of MnO_2_-nonwoven composite air filters was measured in the lab-scale setup (Figure S3). The testing samples were placed in a commercial car air purifier (GP5202, Philips, Lumileds (Shanghai) Management Co., Ltd., Shanghai, China), which was then put in an acrylic reactor (0.232 m^3^). An amount of 0.7 μL of HCHO solution was injected into the reactor, and then the indoor air was blended by a 5 watt fan, which was fixed on top of the reactor. When the concentration of HCHO was stabilized to 0.89 ppm, the car air purifier was switched on to start the test. The concentration of HCHO was monitored by a portable gas detector (BSQ-BCH2O, Shanghai Benshan Instrument and Equipment Co., Ltd., Shanghai, China) at 25 °C for 120 min. The HCHO removal performance was evaluated by plotting (*C*/*C*_0_) versus reaction time, and the reaction rate constant (*k*) was defined as Equation (1):

(1) $$k=\ln\left(\frac{C_{0}}{C}\right)/t$$

where *C*_0_ (ppm) is the initial concentration of HCHO before the test, and *C* (ppm) is the dynamic concentration of HCHO at different time points. 

## 3. Results and Discussion

### 3.1. Production and Characterization of MnO_2_-Nonwoven Composites

**Figure 2:**
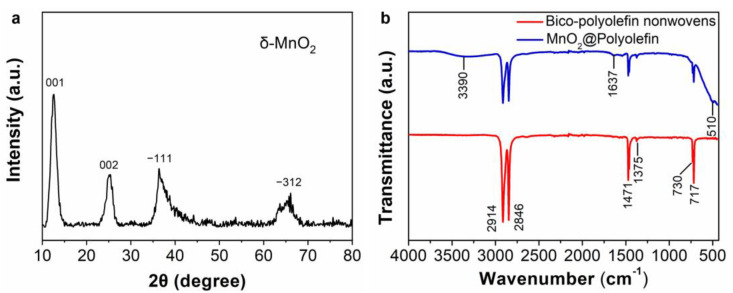
(**a**) XRD pattern of δ-MnO_2_; (**b**) FTIR spectra of bico-polyolefin spunbond nonwovens and MnO_2_@Polyolefin filter.

The crystallographic structure of MnO_2_ crystal was identified by using XRD. Figure 2a presents the main diffraction peaks at 12.5°, 25.1°, 36.1°, and 65.4°, which correspond to the characteristic peaks of δ-MnO_2_ [30].The FTIR spectra show surface features of bico-polyolefin nonwovens and a MnO_2_@Polyolefin sample. As can be seen from the red spectrum of PE/PP nonwovens in Figure 2b, the peaks at 2914 and 2846 cm^−1^ are assigned to the CH_2_ asymmetric stretch and CH_2_ symmetric stretch modes, respectively [31]. Interestingly, the characteristic peaks of low-density polyethylene (LDPE) and high-density polyethylene (HDPE) are all observed on the spectrum of PE/PP nonwovens. The peak located at 1375 cm^−1^ belongs to the CH_2_ umbrella mode of LDPE, whereas the peaks at 730 and 717 cm^−1^ are split CH_2_ rocking peaks of HDPE [31,32]. The above results indicate that both of the HDPE and LDPE chips were added to produce a PE sheath component in bico-polyolefin spunbond nonwoven production. It is worth noting that no peak of the PP component is observed in the FTIR spectrum due to the core–sheath feature of bico-polyolefin nonwovens. Compared with the red curve, the MnO_2_@Polyolefin FTIR spectrum presents additional peaks at 3390, 1637, and 510 cm^−1^, which are assigned to the stretching vibration of -OH groups, -OH bending mode, and characteristic band of layered manganese oxides, respectively [33]. 

**Figure 3:**
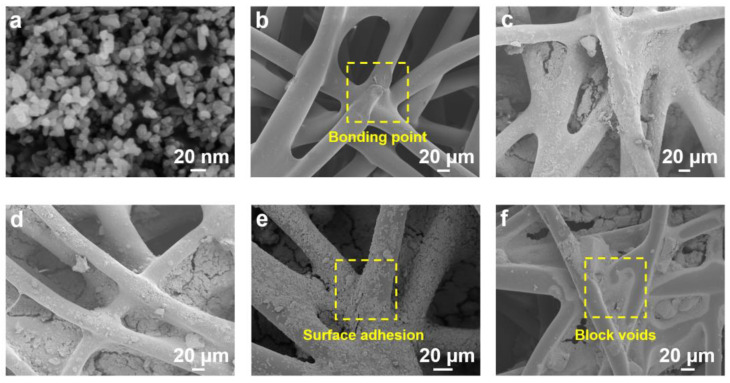
SEM images of (**a**) δ-MnO_2_, (**b**) bico-polyolefin nonwovens, (**c**) 10%MnO_2_@Polyolefin, (**d**) 15%MnO_2_@Polyolefin, (**e**) 20%MnO_2_@Polyolefin, and (**f**) MnO_2_@binder@Polyolefin.

The morphologies of δ-MnO_2_ crystals, bico-polyolefin nonwovens, and MnO_2_-nonwoven composites were investigated by SEM. As shown in Figure 3a, δ-MnO_2_ crystals appear as spherules with an average diameter of 30 nm. The surface of bico-polyolefin nonwovens is relatively smooth compared with the MnO_2_-nonwovens composites. The bonding points are clearly observed due to the diffusion of the molten PE sheath under appropriate temperature [34]. It can be seen from Figure 3c–e that a MnO_2_ nanocrystalline is firmly attached to the fiber surface, where the PE component acts as binders and assists in MnO_2_-fiber adhesion. Notably, when a higher MnO_2_ concentration is prepared in solution, the coverage areas for bico-polyolefin nonwovens increase significantly. It turns out that 20%MnO_2_@Polyolefin samples achieved almost full coverage. In contrast, as shown in Figure 3f, when the SAE adhesive was used to bind MnO_2_, the crystals tended to aggregate on fiber voids. We ascribe the observed particle agglomeration and block to the different surface tension caused by SAE. This is in stark contrast to the binder-free procedure that yields uniform and dense MnO_2_ coatings on the surface of bico-polyolefin spunbond nonwovens.

### 3.2. Evaluation of Pore Size Distribution of Bico-Polyolefin Nonwovens and MnO_2_-Nonwoven Composites

**Figure 4:**
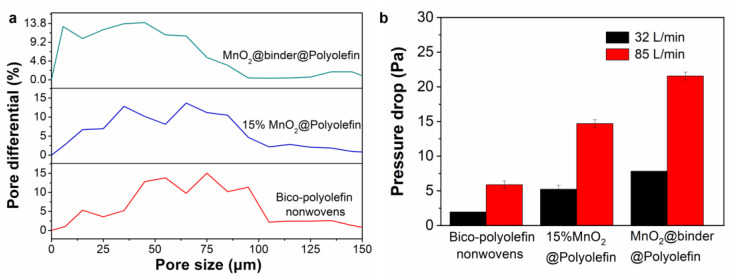
(**a**) Pore size distribution and (**b**) pressure drop of bico-polyolefin nonwovens, 15%MnO_2_@Polyolefin, and MnO_2_@binder@Polyolefin.

The pore size distribution results for bico-polyolefin nonwovens and MnO_2_-nonwoven composites are presented in Figure 4a. The bico-polyolefin nonwoven sample shows disordered pore size distribution as several peaks stand out in profile. The addition of MnO_2_ to the bico-polyolefin nonwovens leads to a narrowed and gradient pore size distribution. Taking 15%MnO_2_@Polyolefin as an example, it reveals three dominant peaks centered in sequence at around 13, 37, and 63 μm. The mean pore diameter decreases from 67.75 to 52.85 μm compared with the bico-polyolefin nonwovens. We observe that the peak becomes broader and shifts for the MnO_2_@binder@Polyolefin sample, which gives rise to a further decreased mean pore diameter value, reaching 41.57 μm. However, using SAC adhesive to bind MnO_2_ did not create a gradient pore distribution for MnO_2_-nonwoven composites because most of the fiber junctions were blocked, which is consistent with the SEM results in Figure 3f. The results in Figure 4b reflect that the marked increase in pressure drop for MnO_2_@binder@Polyolefin is also attributed to the blocked pores, and a decreased mean pore diameter correlates with dropped air permeability.

### 3.3. HCHO Removal Performance of MnO_2_-Nonwoven Composites

**Figure 5:**
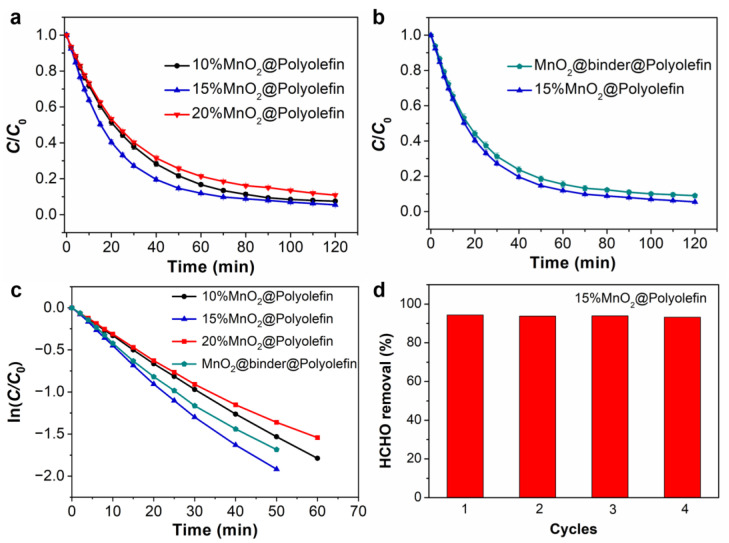
(**a**) HCHO removal test on 10%MnO_2_@Polyolefin, 15%MnO_2_@Polyolefin, and 20%MnO_2_@Polyolefin. (**b**) Comparisons of HCHO removal performance on 15%MnO_2_@Polyolefin and MnO_2_@binder@Polyolefin. (**c**) Reaction kinetic curves of 10%MnO_2_@Polyolefin, 15%MnO_2_@Polyolefin, 20%MnO_2_@Polyolefin, and MnO_2_@binder@Polyolefin for HCHO removal following the pseudo-first-order model. (**d**) Reproducibility tests of HCHO removal performance on 15%MnO_2_@Polyolefin.

A low concentration HCHO removal performance over MnO_2_-nonwoven composites was investigated. Figure 5a shows HCHO removal results for 10%MnO_2_@Polyolefin, 15%MnO_2_@Polyolefin, and 20%MnO_2_@Polyolefin. All samples show good HCHO removal efficiency at low concentrations, and 15%MnO_2_@Polyolefin removed 94.5% ± 0.4% of HCHO within 120 min. Likewise, 10%MnO_2_@Polyolefin and 20%MnO_2_@Polyolefin removed 92.5% ± 0.6% and 89.1% ± 0.7%, respectively, in a same time period. The above results indicate that overloading MnO_2_ crystals on nonwovens hinders the performance of HCHO catalytic removal. As shown in Figure 5b, using binders to produce MnO_2_-nonwoven composites could not only raise environmental concerns, but also lead to worse removal performance. The HCHO removal efficiency for the MnO_2_@binder@Polyolefin sample was reduced by over 4% compared with 15%MnO_2_@Polyolefin. It is believed that particle agglomeration and encapsulation by an adhesive are the main factors that contribute to worse removal performance. In Figure 5c, we plotted the kinetic curves by measuring ln(*C*/*C*_0_) versus the reaction time. According to the pseudo-first-order model, the initial rate constants of 10%MnO_2_@Polyolefin, 15%MnO_2_@Polyolefin, 20%MnO_2_@Polyolefin, and MnO_2_@binder@Polyolefin were 0.030, 0.040, 0.027, and 0.035 min^−1^, respectively. In addition, 15%MnO_2_@Polyolefin exhibited impressive reproducibility (Figure 5d) as the HCHO removal performance showed no significant difference after four tests. The HCHO removal performance of selected catalysts is summarized in Table S1, Supporting Information. In comparison with other materials, 15%MnO_2_@Polyolefin showed acceptable performance in overall HCHO removal.

## 4. Conclusions

In summary, we demonstrated a robust approach to produce binder-free MnO_2_-nonwoven composite filters using δ-MnO_2_ crystals bonded firmly on the surface of bicomponent PE/PP spunbond nonwovens. The SEM images of MnO_2_@Polyolefin samples show that the molten PE component enables MnO_2_ to be attached to a fiber surface, whereas nanocrystalline MnO_2_ tends to be aggregate at fiber junctions to block inherent pores for MnO_2_@binder@Polyolefin. Results of pore size distribution and pressure drop confirm that the gradient porous structures are constructed for MnO_2_@Polyolefin filters. Moreover, the HCHO removal test elucidates that binder-free MnO_2_@Polyolefin filters show improved HCHO removal performance (94.5% ± 0.4%, *k* = 0.040 min^−1^) in contrast to MnO_2_@binder@Polyolefin filters (90.2% ± 0.9%, *k* = 0.035 min^−1^). We believe that such a green and environmentally friendly approach will provide an alternative solution in producing catalyst/fabric composite filters.

## Figures and Tables

**Table 1:** Preparation details in MnO_2_-nonwoven composites.

Sample	Concentration of MnO_2_ in Precursor Solution	MnO_2_ Content in MnO_2_-Nonwoven Composite
10%MnO_2_@Polyolefin	10%	45% ± 3%
15%MnO_2_@Polyolefin	15%	54% ± 4%
20%MnO_2_@Polyolefin	20%	66% ± 4%
MnO_2_@binder@Polyolefin	15%	50% ± 3%

## Data Availability

Not applicable.

## Supplementary Materials

The following supporting information can be downloaded at: https://www.mdpi.com/article/10.3390/polym14122504/s1, Figure S1: Washing step to remove PVA and unbonded MnO_2_ crystals; Figure S2: Optical photograph of bico-polyolefin nonwovens and 15%MnO_2_@Polyolefin; Figure S3: Lab-scale setup for formaldehyde removal testing; Figure S4: Linear fitting of the reaction kinetic curves; Table S1: Summary of HCHO removal performance on a selected catalyst.
