# Supplementary material for: Gradient Porous Structured MnO2-Nonwoven Composite: A Binder-Free Polymeric Air Filter for Effective Room-Temperature Formaldehyde Removal
Source: Polymers (Basel). 2022 Jun 20;14(12):2504. doi: 10.3390/polym14122504 (PMC9231320; doi:10.3390/polym14122504)
Supplement: Supplementary file 1 [file polymers-14-02504-s001.zip › polymers-1755764-SI.pdf]

Supplementary Materials

Gradient porous structured MnO_2-nonwoven composite: A binder-free polymeric air filter for effective room-temperature formaldehyde removal

Zijian Dai [1, 2], Jianyong Yu [1, 2,] and Yang Si [1,*]*

1; State Key Laboratory for Modifcation of Chemical Fibers and Polymer Materials College of Materials Science and Engineering Donghua University, Shanghai 201620, China zjdai@dhu.edu.cn (ZD)

2 Innovation Center for Textile Science and Technology, Donghua University, Shanghai 200051, China

* Correspondence: yujy@dhu.edu.cn (JY); yangsi@dhu.edu.cn (YS)

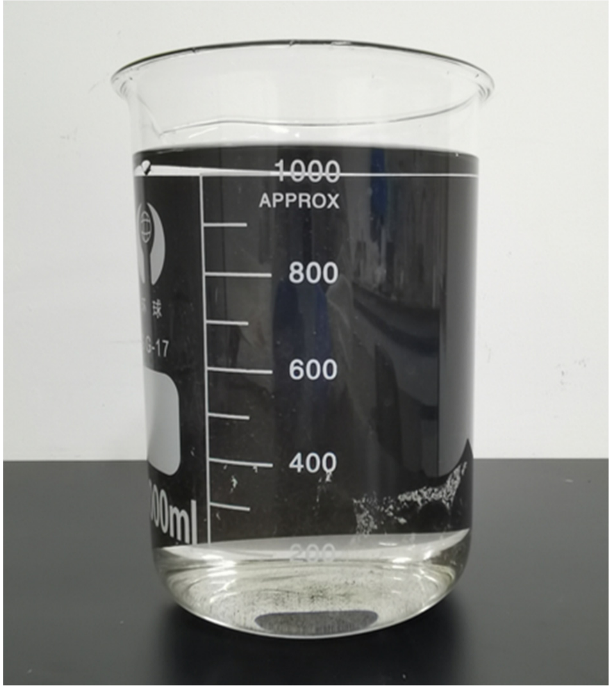

Figure S1. Washing step to remove PVA and unbonded MnO_2 crystals.

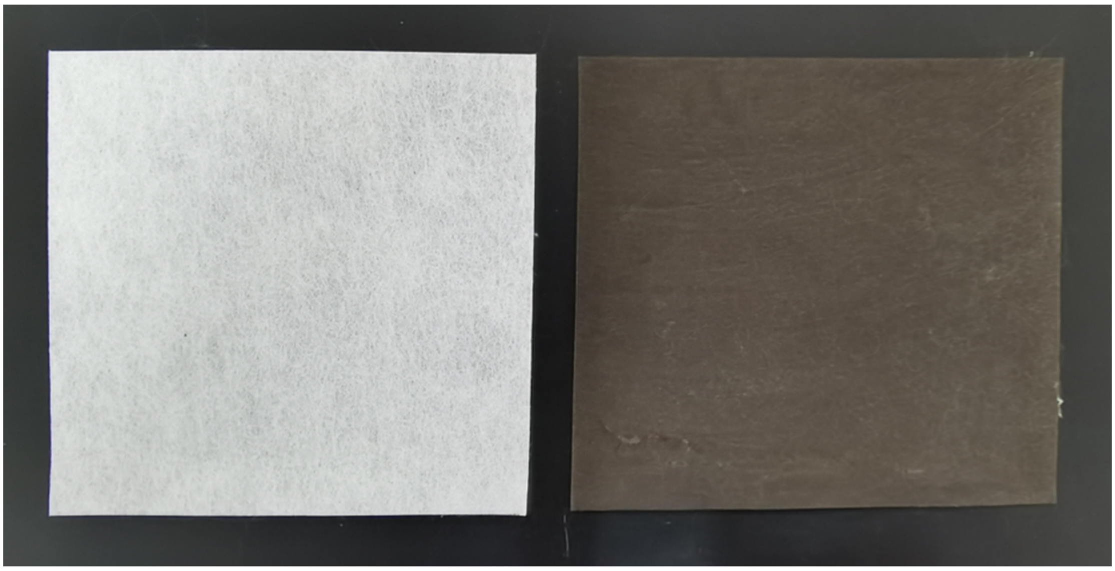

Figure S2. Optical photograph of bico-polyolefin nonwovens and 15%MnO_2@Polyolefin.

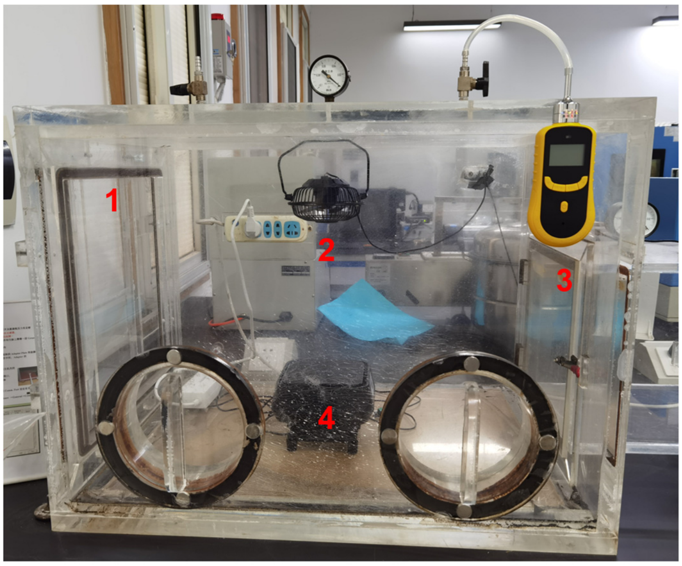

1-Acrylic reactor; 2-Fan; 3-Portable gas detector; 4-Car air purifier

Figure S3. Lab-scale setup for formaldehyde removal testing.

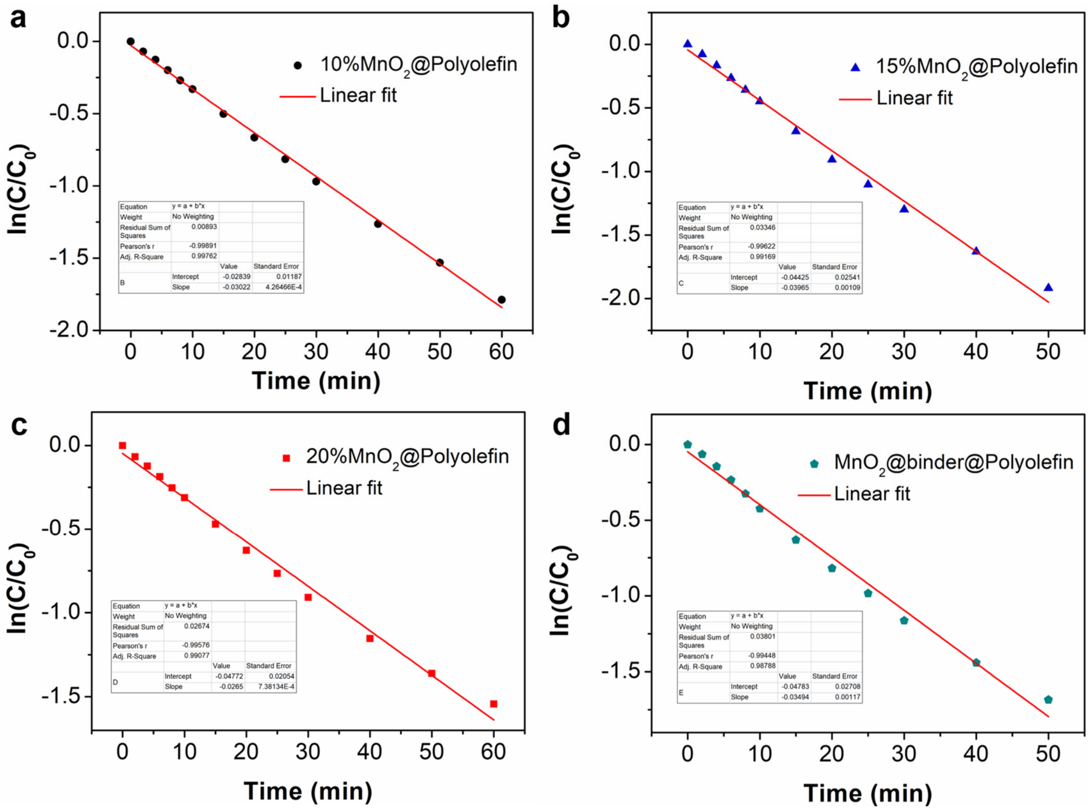

Figure S4. Linear fitting of the reaction kinetic curves.

Table S1 Summary of HCHO removal performance over selected catalyst.

Catalyst	Condition	Reaction rate constant (k)	Reference
BiOCl/Clinoptilolite composite	50 mg/^{3} HCHO; 1g catalyst<br>Chamber volume = 60L<br>Light intensity is 50mW/cm^2	0.0166 min^{-1}	[1]
Bi_2MoO_6/attapulgite	50 mg/^{3} HCHO; 1g sample<br>Chamber volume didn’t report<br>150 W visible light source	0.0109 min^{-1}	[2]
gold-assisted $ZnSn(OH)_6$ microcubes	1ppm HCHO 0.1g sample<br>Chamber volume didn’t report<br>300 W Xenon lamp	~0.25 min^{-1}	[3]
TiO_2-BiOBr-sepiolite composite	80ppm HCHO; 1g sample<br>Chamber volume didn’t report<br>150 W visible light source	0.01574 min^{-1}	[4]
10%MnO_2@Polyolefin	0.89 ppm HCHO ~0.75 g catalyst<br>Chamber volume = 0.232 m^3;	0.03 min^{-1}	This work
15%MnO_2@Polyolefin	0.89 ppm HCHO ~0.6 g catalyst<br>Chamber volume = 0.232 m^3;	0.04 min^{-1}	This work

Reference

1. Di, Y.; Zhang, X.; Wang, X.; Zheng, S., Construction of BiOCl/Clinoptilolite Composite Photocatalyst for Boosting Formaldehyde Removal. *Materials* **2021,** 14, (21).
2. Tan, Y.; Yin, C.; Zheng, S.; Di, Y.; Sun, Z.; Li, C., Design and controllable preparation of Bi2MoO6/attapulgite photocatalyst for the removal of tetracycline and formaldehyde. *Applied Clay Science* **2021,** 215, 106319.
3. Pham, M.-T.; Bui, D.-P.; Lin, I.-F.; Phuong, N. H.; Huang, Y.; Cao, J.; You, S.-J.; Wang, Y.-F., Enhanced near-visible-light photocatalytic removal of formaldehyde over Au-assisted ZnSn(OH)6 microcubes. *Environmental Technology & Innovation* **2020,** 20, 101112.
4. Hu, X.; Li, C.; Song, J.; Zheng, S.; Sun, Z., Multidimensional assembly of oxygen vacancy-rich amorphous TiO2-BiOBr-sepiolite composite for rapid elimination of formaldehyde and oxytetracycline under visible light. *Journal of Colloid and Interface Science* **2020,** 574, 61-73.
